# The association between prehospital vital signs of children and their critical clinical outcomes at hospitals

**DOI:** 10.1038/s41598-022-09271-0

**Published:** 2022-03-25

**Authors:** Hiroshi Kurosawa, Yuko Shiima, Chisato Miyakoshi, Mari Nezu, Maki Someya, Minae Yoshida, Hiroaki Nagase, Kandai Nozu, Yoshiyuki Kosaka, Kazumoto Iijima

**Affiliations:** 1 Department of Pediatrics, Kobe University Graduate School of Medicine, Hyogo, Japan; 2 Department of Advanced Pediatric Medicine, Kobe University Graduate School of Medicine, Hyogo, Japan; 3 Division of Pediatric Critical Care Medicine, Hyogo Prefectural Kobe Children’s Hospital, 1-6-7, Minatojima-Minamimachi, Chuo-ku, Kobe, Hyogo 650-0047 Japan; 4 Department of Research Support, Center for Clinical Research and Innovation, Kobe City Medical Center General Hospital, Hyogo, Japan; 5 Department of Pediatrics, Kobe City Medical Center General Hospital, Hyogo, Japan

**Keywords:** Paediatric research, Paediatrics, Respiratory signs and symptoms

## Abstract

Vital signs are important for patient assessment, but little is known about interpreting those of children in prehospital settings. We conducted an observational study to investigate the association between prehospital vital signs of children and their clinical outcomes in hospitals. We plotted the data of patients with critical outcomes on published reference ranges, such as those of healthy children to evaluate the clinical relevance. Of the 18,493 children screened, 4477 transported to tertiary hospitals were included in the analysis. The outcomes 12 h after being transported to a tertiary hospital were as follows: deceased, 41; hospitalization with critical deterioration events, 65; hospitalization without critical deterioration events, 1086; returned home, 3090; and unknown, 195. The reference ranges of the heart rates (sensitivity: 57.7%, specificity: 67.5%) and respiratory rates (sensitivity: 54.5%, specificity: 67.7%) of healthy children worked best to detect the critical outcomes. Therefore, the reference ranges of healthy children were concluded to be suitable in prehospital settings; however, excessive reliance on vital signs carried potential risks due to their limited sensitivities and specificities. Future studies are warranted to investigate indicators with higher sensitivities and specificities.

## Introduction

Vital signs are among the most important factors for pediatric patient assessment. These include the evidence-based centile curves of heart rates (HRs) and respiratory rates (RRs) of the following: healthy children^1^, children in emergency departments^2,3^, hospitalized children^4^, and hospitalized critically ill children (HRs and blood pressure centile curves)^5^. These charts are widely referred to for patient assessments^6,7^ and pediatric triage scales in emergency departments^3,8–11^. However, the reliability and validity of the pediatric triage scales are insufficient^12–14^. Some recent triage scales in emergency departments have incorporated the charts of healthy children; however, they still require modifications to ensure patient safety and appropriate dispositions^8,9,11,15^.

The interpretation of vital signs is more challenging in prehospital settings as compared to in the emergency departments. This may be due to various factors that affect the pediatric vital signs, such as uncomfortable and stressful environments (being surrounded by strangers, environmental temperature, noise, etc.) and physiological stressors (uncontrolled pain, convulsion, fever, etc.). Emergency medical service providers are not comfortable with assessing and transporting children^16,17^. Some emergency transport systems, including the one in Kobe City (Kobe City Emergency Transport System, Japan), use a triage scale (which incorporates vital signs) to decide on the destination hospitals; however, to the best of our knowledge, such scales are not validated. Understanding the meaning of vital signs is critical for allowing emergency medical service providers to transport children to a suitable hospital.

Thus, this study aimed to investigate the associations between the vital signs of children in prehospital settings and their outcomes at hospitals using a modified critical deterioration metric. It also aimed to evaluate the usefulness of published centile curves, such as those for healthy children, children in emergency departments, and hospitalized children in prehospital settings.

## Results

### Characteristics of the study participants

**Figure 1:**
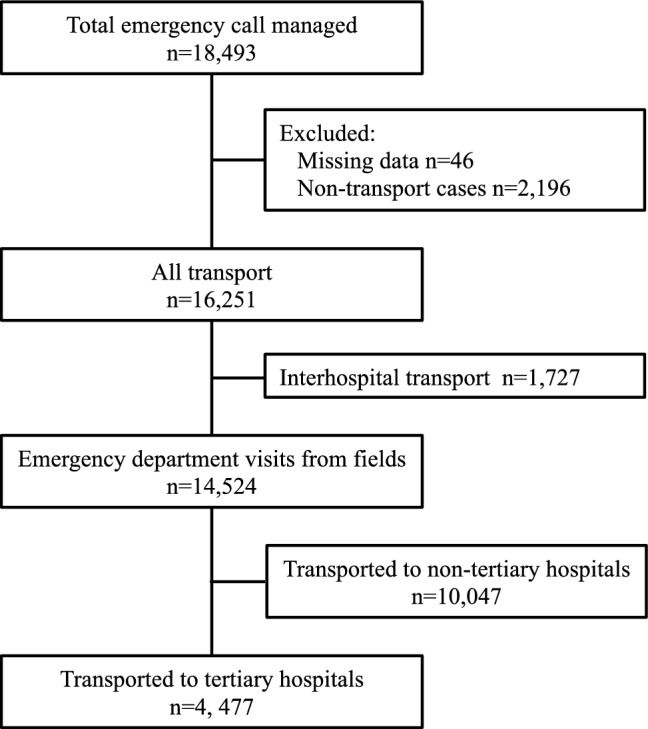
Study population.

Overall, 18,493 patients aged < 19 years were registered in the Kobe City Emergency Transport System during the study period. They were transported to approximately 160 hospitals, including eight tertiary hospitals. We excluded 2242, 1727, 14,524 patients due to non-transport or missing data, transfers between hospitals, and transfers to non-tertiary hospitals, respectively. Therefore, 4477 patients were included in the analysis (Fig. 1). The median age was 4 years (interquartile range, 1–11 years), and 2667 (59.6%) were male. The median transport time from the scene to the hospital was 13 min (interquartile range, 8–18 min).

The HRs and RRs were recorded at least once either when the patient was loaded into the ambulance or at the scene in 4225 and 3328 patients, respectively. Furthermore, 96.8% (4091/4225) and 78.0% (2597/3328) of the HR and RR measurements were performed when the patients were loaded into the ambulance, respectively. Moreover, 3.2% (134/4225) and 22.0% (731/3328) of the HR and RR measurements were performed at the scene, respectively. The standardized mean differences of the two data points were 0.012 (HR) and 0.052 (RR).

**Figure 2:**
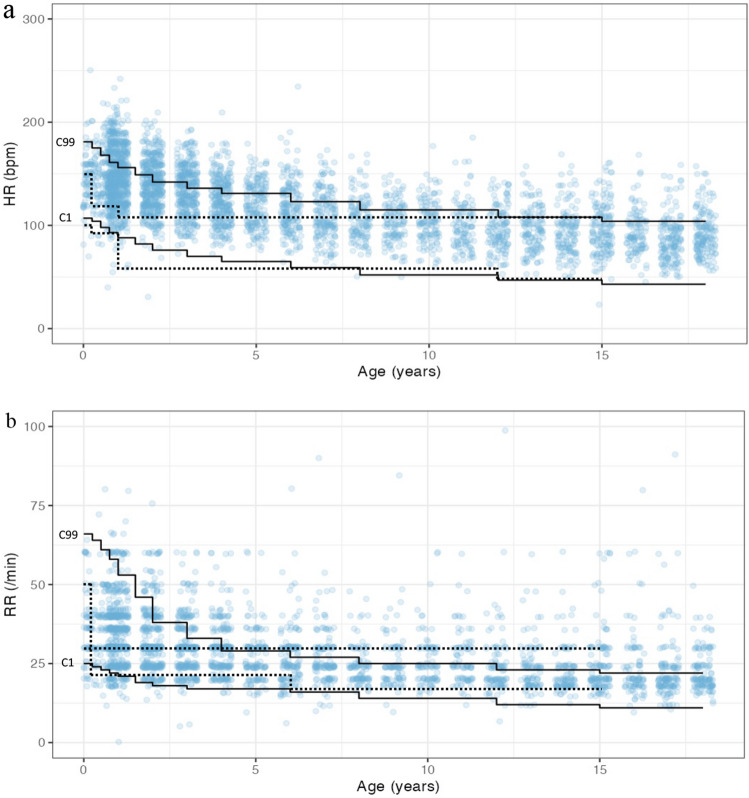
Centile curves of the heart rates (**a**) and respiratory rates (**b**) of healthy children as developed by Fleming et al.^1^ (solid lines), reference ranges of the triage scale of the Kobe City Emergency Transport System (dotted lines), and plots of the study population. C1, the 1st centile; C99, the 99th centile.

Figure 2 demonstrates the reference ranges of healthy children, the reference ranges used by the Kobe City Emergency Transport System, and the plots of the study population.

Critical deterioration events occurred in 65 patients; among these, requirement of non-invasive ventilation, invasive mechanical ventilation, and vasopressor infusion was noted in 11, 53, and 23 patients, respectively.

### Main results

**Figure 3:**
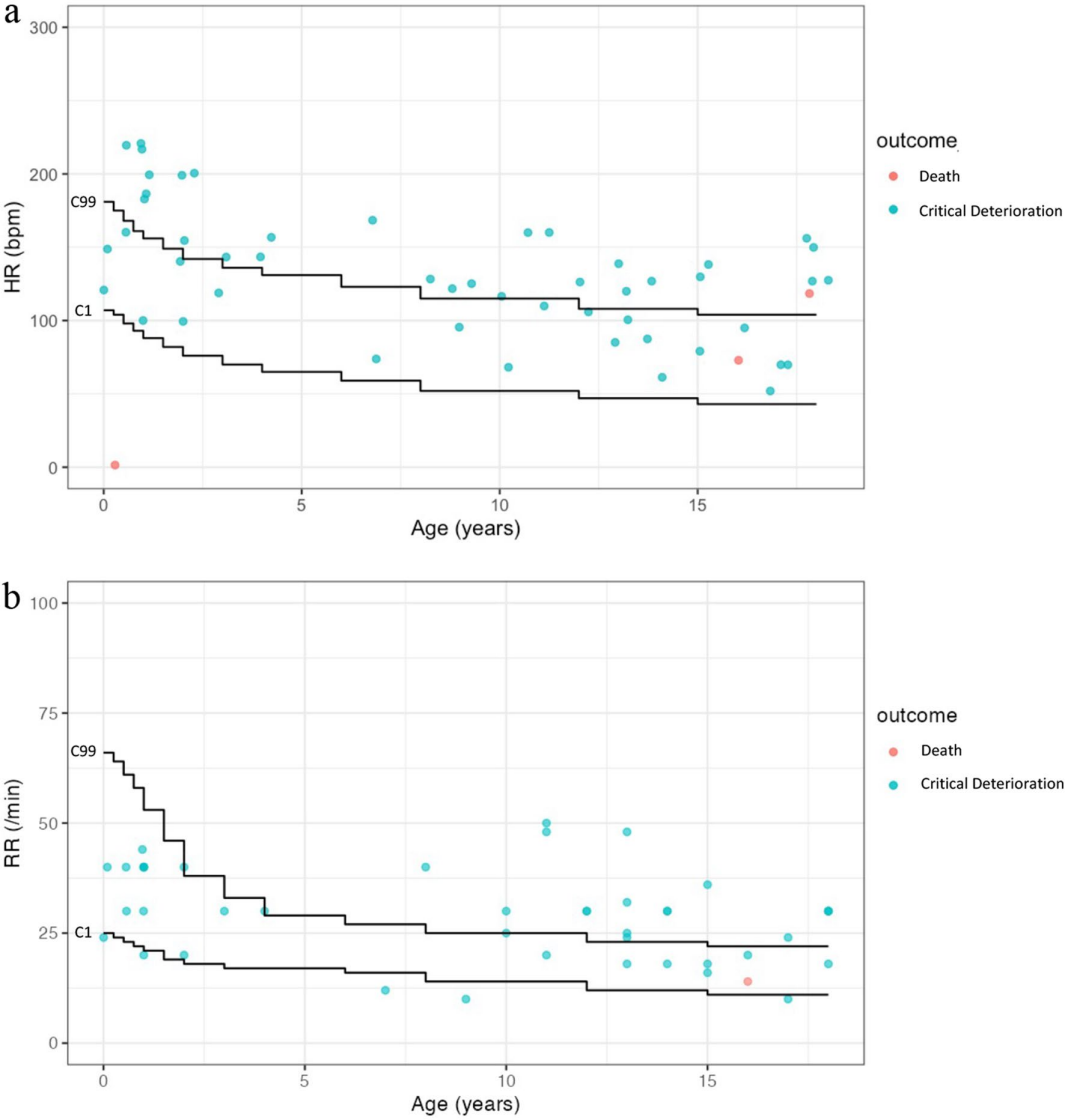
Centile curves of the heart rates (**a**) and respiratory rates (**b**) of healthy children as developed by Fleming et al.^1^ and plots of patients who died or were hospitalized with critical deterioration events. Data on patients without vital signs recorded at the two time points by the emergency medical service providers are not displayed here. Critical Deterioration, hospitalized with critical deterioration events; C1, the 1st centile; C99, the 99th centile.

When plotting the vital sign data of patients with critical outcomes on the centile curves of healthy children, the HRs and RRs were outside of the 1st and 99th centiles for 30/52 (58%) and 24/44 (55%) patients, respectively (Fig. 3). The number of patients outside the range was larger when the centile curves of healthy children were used as compared to when the centile curves of the children visiting the emergency departments (HR 21/52 [40%] and RR 18/44 [41%]) (Supplementary Fig. S1) and of hospitalized children (HR 15/52 [29%] and RR 7/44 [16%]) (Supplementary Fig. S2) were used. The sensitivity and specificity of the vital signs for the identification of patients with critical outcomes are shown in Table 1.

**Table 1:** The sensitivity and specificity for detecting patients with critical outcomes by using the 1st and 99th centiles of each reference range of the vital signs from the previously developed centiles, as well as the reference ranges of the Kobe City Emergency Transport System.

	Heart rate		Respiratory rate	
	Sensitivity (%) [95% CI]	Specificity (%) [95% CI]	Sensitivity (%) [95% CI]	Specificity (%) [95% CI]
Healthy children^1^	57.7 [44.3; 71.1]	67.5 [66.1; 69.0]	54.5 [39.8; 69.3]	67.7 [66.1; 69.3]
Children at emergency departments^2^	40.4 [27.0; 53.7]	85.7 [84.6; 86.8]	40.9 [26.4; 55.4]	77.6 [76.2; 79.1]
Hospitalized children^4^	28.8 [16.5; 41.2]	91.0 [90.1; 91.9]	15.9 [5.1; 26.7]	95.2 [94.5; 96.0]
Kobe City Emergency Transport System	67.3 [54.6; 80.1]	40.6 [39.1; 42.1]	43.2 [28.5; 57.8]	71.4 [69.8; 72.9]

CI, confidence interval.

Among the patients transported to tertiary hospitals, 41 died within 12 h after transport; all of these had been assessed as deceased or critical by the emergency medical service providers. Furthermore, 62% (40/65) of the hospitalized patients with critical deterioration events were assessed as critical or severe by the emergency medical service providers (Supplementary Table S1). The sensitivity and specificity of the emergency medical service assessments of severity were 76% (81/106) and 98% (4088/4176), respectively.

## Discussion

Our findings suggested that prehospital vital signs may help identify half of the pediatric patients with critical deterioration events when the centiles of healthy children, which had the highest sensitivity and reasonably high specificity among the centiles for vital signs that were developed previously, were used as the reference ranges (Table 1). In prehospital settings, where time for patient assessment is limited, high sensitivity is more important than specificity to avoid preventable pediatric death. The study results may be informative for emergency medical service providers in their measurement and interpretation of the vital signs of pediatric patients. Moreover, even when the vital signs were within the 1st and 99th centiles of those in healthy children, critically ill children were latent; therefore, caution must be exercised during the assessment. Our study findings suggest that it may be difficult to develop vital sign reference ranges with high sensitivities and specificities.

When we plotted the findings of the study population (4477 children) according to the reference range used by the Kobe emergency medical service, the majority of the cases were outside the range. This was because the ranges were too narrow, especially for younger age groups (Fig. 2); this resulted in high sensitivities but low specificities of the HRs. The scales were defined by local experts, and the current study suggested that it would be appropriate to update these scales, because an excessively low specificity may result in excessive over-triages of children and an inappropriate utilization of resources in the tertiary hospitals.

The emergency medical service providers in Kobe City, however, conducted triages well, given the high sensitivity and specificity of their judgment. While they might have been puzzled by the narrow “normal” ranges of the vital signs, their down-triage was appropriate. It was not clear from the study how the emergency medical service providers down-triaged patients who had “abnormal” vital signs, and future studies on this are warranted.

This study had several limitations. First, the data were obtained retrospectively from a database of one city; therefore, our findings may not be generalizable. However, the large sample size, comprehensive assessments by the emergency medical services, and the outcomes defined using the modified critical deterioration metric make the study finding valuable. Second, emergency medical service providers in Japan are allowed a limited range of interventions; therefore, the study findings are not directly applicable to regions where other interventions are allowed in prehospital settings. Third, we utilized vital signs at two points, i.e., when the patient was loaded into the ambulance and at the scene. However, the standardized mean differences of the two data points were minimal. Fourth, we did not investigate the long-term patient outcomes. Instead of the long-term outcomes, the modified critical deterioration metric may have reflected the prehospital conditions more accurately, with minimal effects of the interventions after transport to hospitals. Lastly, we could not investigate the outcomes at non-tertiary hospitals in the database, which also limits the generalizability of the study findings. A recent study analyzed patients from the same database in Kobe City, who were secondary-transported to tertiary hospitals^18^. The study showed that 81 patients were transported to non-tertiary hospitals, and then secondary-transported to a tertiary hospital within 24 h of the first transport during the same study period. One died and eight were hospitalized and required critical care. We conducted a sensitivity analysis to detect patients with critical outcomes, including those who were transported to a non-tertiary hospital and then secondary-transported to a tertiary hospital (Supplementary Table S2).

In summary, the vital signs in prehospital settings may be used to detect half of the severely ill children who may die or be hospitalized with critical deterioration events at 12 h after emergency transport. The use of the centiles of healthy children as prehospital vital sign reference ranges may be suitable; however, excessive reliance on vital signs carries potential risks, because their sensitivities and specificities are limited. Future studies are warranted to investigate the indicators with higher sensitivities and specificities.

## Methods

### Data sources and setting

We conducted this retrospective observational study using the Kobe City Emergency Transport System database. Kobe is the seventh-largest city in Japan and has a population of approximately 1.5 million; this includes 250,000 children aged < 19 years. The Kobe emergency transport system covers 550 km^2^ and has 30 emergency medical service stations. The emergency medical service in Japan has unique features, with a strictly limited range of interventions allowed for use by the emergency medical service providers. The emergency medical service providers in Kobe are trained in basic life support and are allowed to administer intravenous fluids to patients of cardiac arrest. However, they are neither allowed to intubate patients ≤ 14 years of age nor allowed to administer adrenaline to patients ≤ 7 years of age. Although providing oxygen and performing bag-mask ventilation are permitted, they are not allowed to administer opioids or other analgesics, antipyretics, or anti-epileptic medications. Therefore, in Kobe City, the change in vital signs during prehospital transport might be minimal due to the limited interventions allowed.

The database comprises records of all cases in which emergency medical services were dispatched, regardless of whether the patients were transported to hospitals or not. We included children < 19 years of age who were transported to hospitals by the Kobe City Emergency Transport System between January 2013 and December 2015. Patients who were transferred between hospitals were excluded because the vital signs recorded by the emergency medical service providers in such cases were subject to fluctuation due to interventions that were implemented at the referring hospital. Patients who were not transported from the field were also excluded. Supplementary Table S3 shows the differences in the vital signs between patients who were not transported and those who were transported. We also excluded patients who were transported to non-tertiary hospitals, because investigating their outcomes was not feasible. Supplementary Fig. S3 shows the centile curves of the vital signs for patients transported to tertiary hospitals and non-tertiary hospitals to overview the vital signs of all pediatric patients in the database.

We extracted data on the vital signs, age, sex, severity of the patient’s condition (as assessed by the paramedics), time from the call for an ambulance to the arrival at the scene, time from the departure from the scene to the arrival at the hospital, and name of the hospital where the patients were transported to. The vital signs were recorded by the paramedics at the following time points: (i) when they contacted the patient at the scene, (ii) when they loaded the patient into the ambulance, and (iii) when they arrived at the hospital. Generally, the HRs were obtained from the electrocardiogram readings; however, pulse rates were also recorded sometimes, depending on the situation. The RRs were measured by auscultation using a stethoscope.

The destination hospitals were selected by the emergency medical service providers according to local protocols (Supplementary Fig. S4); these protocols were determined by the age, vital signs, symptoms, and mechanism of injuries of the trauma patients. The prehospital severity categories were assessed by the emergency medical service providers and comprised the following: mild (no hospitalization expected), moderate (not severe, but hospitalization expected), severe (life-threatening), critical (impending life crisis, such as cardiac arrest, respiratory arrest, or requirement of cardiopulmonary resuscitation), and deceased (death at the time of contact).

### Data preparation

We excluded the vital signs recorded during and after cardiopulmonary resuscitation. Although each patient could have two or more observations, we used only one value for the HR and RR in the following order of priority: recorded when the patient was loaded into the ambulance and at the scene. The ECG monitor was attached when a patient was loaded into the ambulance; it was predicted to yield the most accurate initial value. RRs were determined by auscultation at the scene and were not necessarily re-examined when a patient was loaded into the ambulance. Therefore, we utilized both data points. We did not use the values of the HRs and RRs recorded on arrival at hospitals due to the potential influence of environmental or temporal factors, even though the emergency medical service providers in Japan are allowed very limited interventions. We assessed the changes in the two data points used for the vital signs by analyzing the standardized mean differences in order to support this rationale.

### Outcomes

We collected data on patient outcomes at hospitals by reviewing the medical records of critically ill children at eight tertiary hospitals in Kobe City. We defined the patient outcomes at hospitals using a modified critical deterioration metric because deaths in the pediatric population are expected to be rare. The original critical deterioration metric was defined as receiving life-sustaining interventions 12 h after transfer to the intensive care unit (ICU), which was developed and validated in the context of the pediatric rapid response system^19,20^. Life-sustaining interventions included the initiation of non-invasive ventilation, invasive mechanical ventilation, and vasopressor infusion administration (i.e., administration of dobutamine, dopamine, adrenaline, isoproterenol, milrinone, or noradrenaline). We defined the modified critical deterioration metric as receiving life-sustaining interventions 12 h after prehospital transport rather than after ICU admission. We included children who were not transferred to the ICU because the ICU admission criteria were different among the hospitals, and the critical deterioration events could also occur outside the ICU. We defined the critical outcome as deceased within 12 h or hospitalized with critical deterioration events at 12 h after transport.

To evaluate the clinical relevance, we compared the values of patients with critical outcomes with the corresponding reference ranges published by Fleming et al.^1^ for healthy children, O’Leary et al.^2^ for children attending the emergency department, and Bonafide et al.^4^ for hospitalized children. When calculating the sensitivity and specificity for detection of patients with critical outcomes, we used the 1st and the 99th percentiles of the reference range of each vital sign from the previously developed centiles, as well as the reference ranges used by the Kobe City Emergency Transport System.

For validating the severity assessment by the emergency medical service providers, sensitivity was defined as the percentage of patients who were assessed as deceased, critical, or severe by the providers; the denominator comprised hospital outcomes of death or hospitalization with critical deterioration events.

The characteristics and outcomes of the patients are summarized as numbers and percentages for the categorical variables and as medians and interquartile ranges for the continuous variables.

### Ethics

This study was approved by the Institutional Review Board of the Hyogo Prefectural Kobe Children’s Hospital. The study was conducted in accordance with the principles of the Declaration of Helsinki. The need for informed consent was waived because of the retrospective design of the study. All the phases of this study were supported by the Foundation for Ambulance Service Development in Japan. The sponsor did not play a role in the study design, data collection, data analysis, data interpretation, or writing of the manuscript.

## Supplementary Information

Supplementary Information.

## Data Availability

The datasets generated during and/or analyzed during the current study are available from the corresponding author on reasonable request.
