# Supplementary material for: The association between prehospital vital signs of children and their critical clinical outcomes at hospitals
Source: Sci Rep. 2022 Mar 25;12:5199. doi: 10.1038/s41598-022-09271-0 (PMC8956615; doi:10.1038/s41598-022-09271-0)
Supplement: Supplementary file 1 — Supplementary Information. [file 41598_2022_9271_MOESM1_ESM.pdf]

Supplementary Table S1. Prehospital severity judged by emergency medical service providers and hospital outcomes 12 h after the transport using the modified critical deterioration events.

	Deceased	Hospitalized with CDE	Hospitalized without CDE	Returned home	Unknown	Total
Deceased	13	0	0	0	2	15
Critical	28	19	1	0	11	59
Severe	0	21	74	13	12	120
Moderate	0	21	563	584	101	1,269
Mild	0	4	448	2,493	69	3,014
Total	41	65	1,086	3,090	195	4,477

CDE, critical deterioration events

Supplementary Table S2. The sensitivity and specificity to detect patients with critical outcomes including those who were transported to a non-tertiary hospital, and then secondary-transported to a tertiary hospital by using the 1st and 99th centiles of each reference range of vital signs for the previously developed centiles, and the reference ranges of the Kobe City Emergency Transport System.

	Heart rate		Respiratory rate	
	Sensitivity (%)	Specificity (%)	Sensitivity (%)	Specificity (%)
Healthy children[1]	57.7	70.4	54.5	69.2
Emergency department [2]	40.4	87.9	40.9	79.0
Hospitalized children [4]	28.8	92.9	15.9	94.6
Kobe City Emergency Transport System	67.3	48.5	43.2	74.9

Supplementary Table S3. Differences in vital signs between patients who were not transported and those who were transported.

	Not transported	Transported	P-value*
Heart rate at the scene, n	914	4,216	
Median (IQR)	101 (87-120)	110 (90-139)	<0.001
Respiratory rate at the scene, n	933	7,065	
Median (IQR)	24 (20-30)	24 (20-30)	<0.001
Heart rate in the ambulance, n	661	14,799	
Median (IQR)	106 (90-127)	115 (92-140)	<0.001
Respiratory rate in the ambulance, n	279	8,315	
Median (IQR)	24 (20-30)	25 (20-30)	<0.001

IQR, interquartile range.

*Wilcoxon rank-sum test.

Supplementary Figure S1. Centile curves of heart rate (a) and respiratory rate (b) of children visiting the emergency department developed by O'Leary et al.[2] and patients who died or were hospitalized with critical deterioration events. Patients without vital signs recorded during transport were not displayed here. Critical Deterioration, hospitalized with critical deterioration events; C1, the 1st centile; C99, the 99th centile.

a

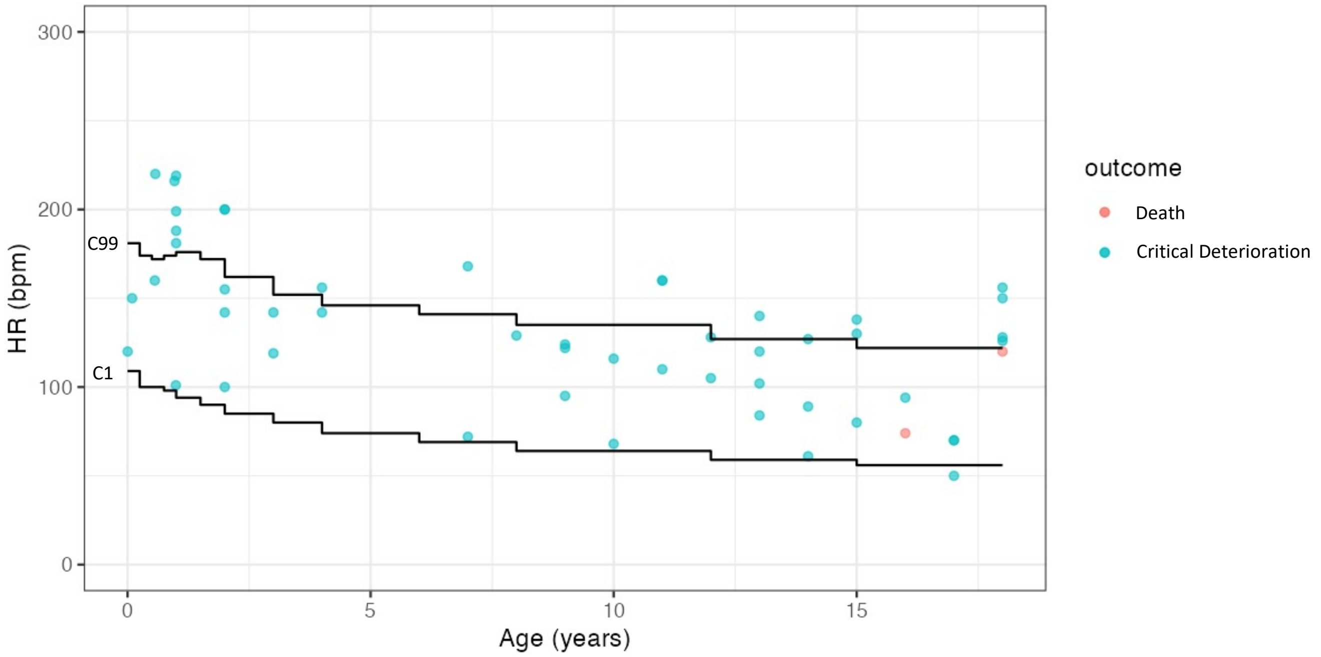

b

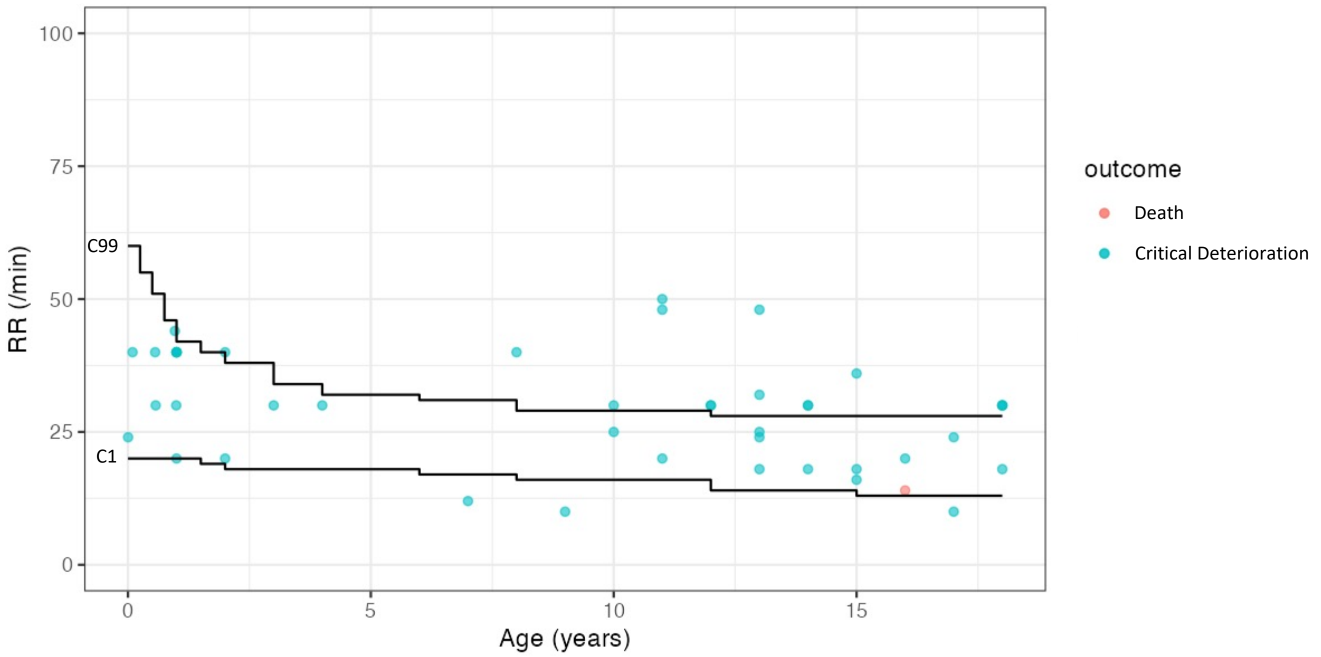

Supplementary Figure S2. Centile curves of heart rate (a) and respiratory rate (b) of hospitalized children developed by Bonafide et al. [4] and patients who died or were hospitalized with critical deterioration events. Patients without vital signs recorded at the two data points by emergency medical service providers were not displayed here. Critical Deterioration, hospitalized with critical deterioration events; C1, the 1st centile; C5, the 5th centile; C95, the 95th centile; C99, the 99th centile.

a

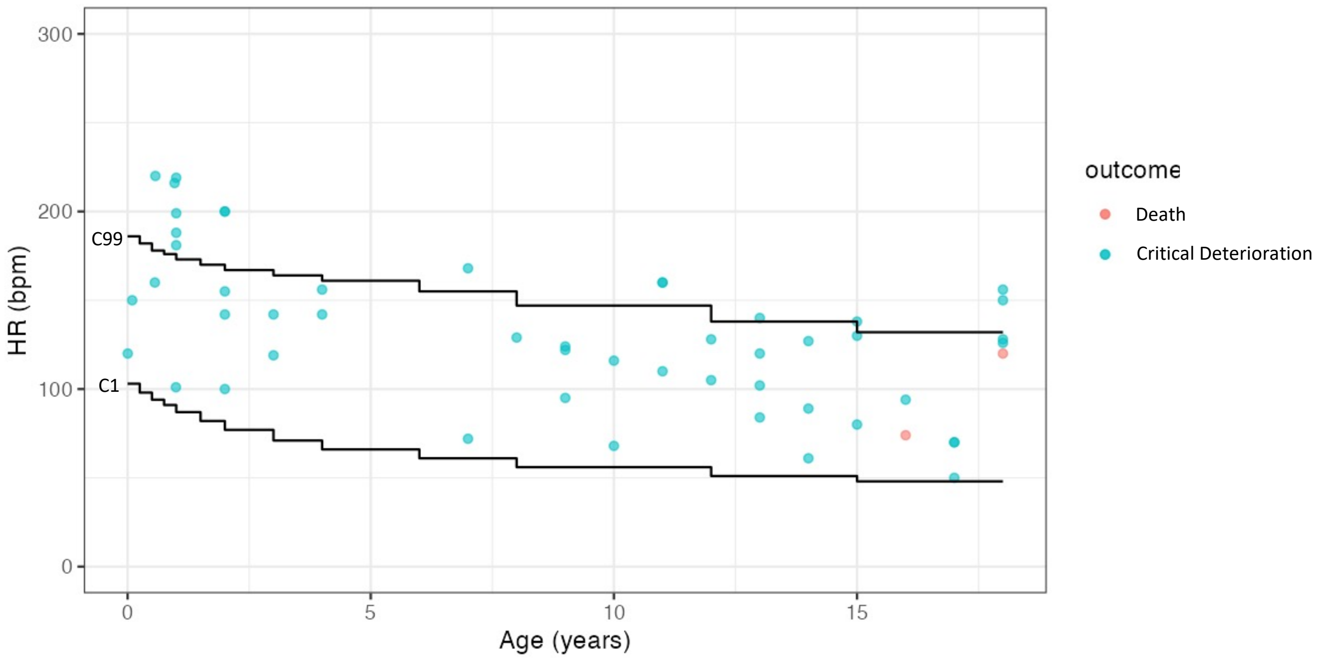

b

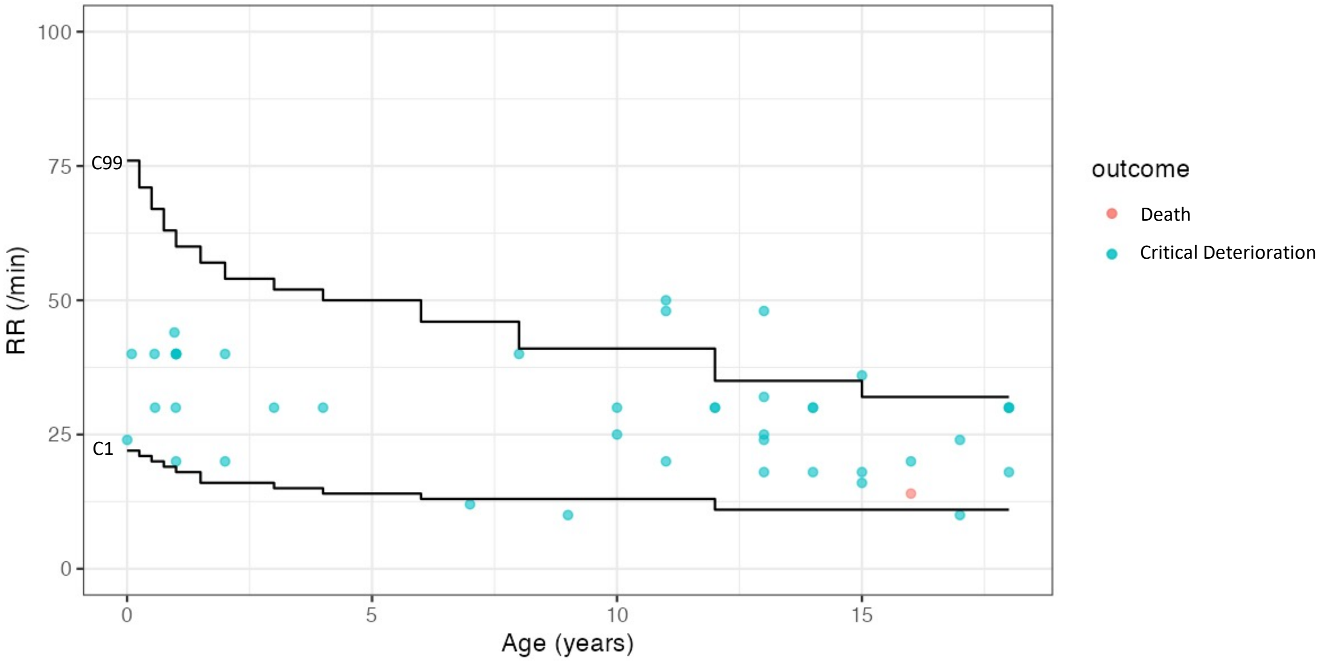

Supplementary Figure S3. Centile curves of heart rates (a) and respiratory rates (b) of the study population. C1, the 1st centile; C5, the 5th centile; C50, the 50th centile; C95, the 95th centile; C99, the 99th centile.

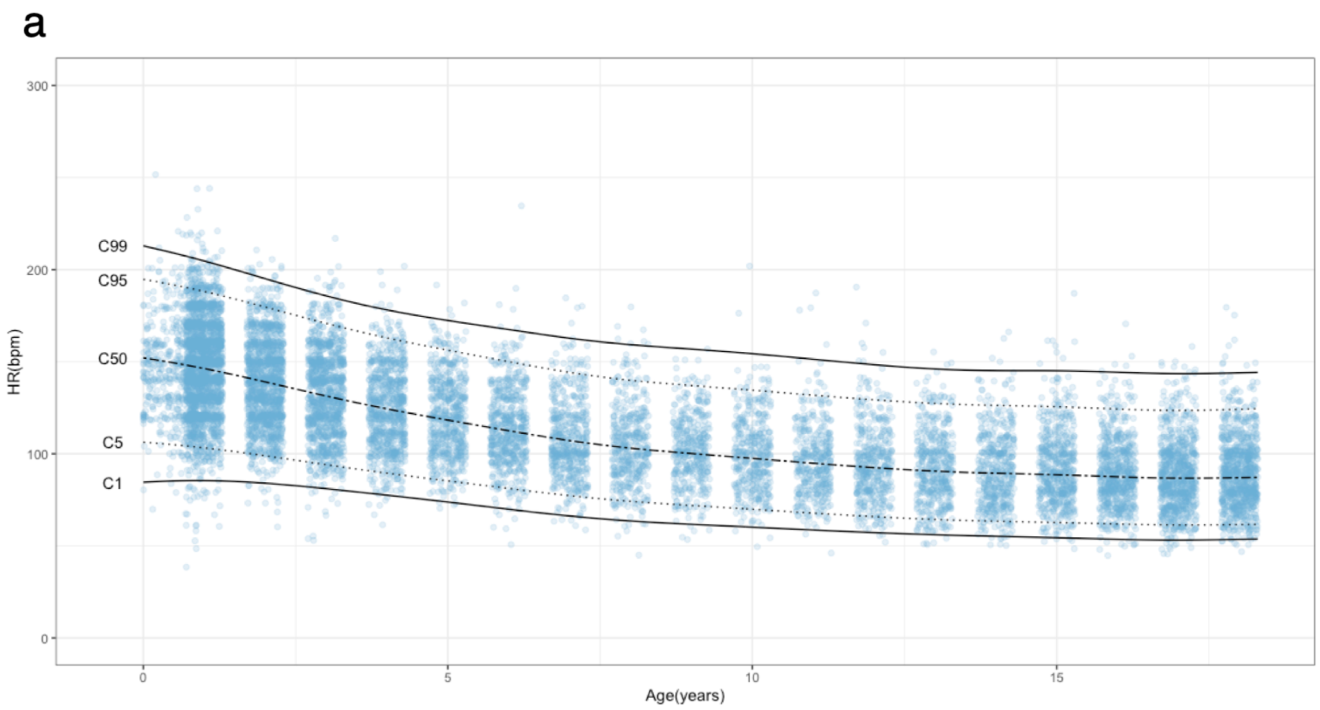

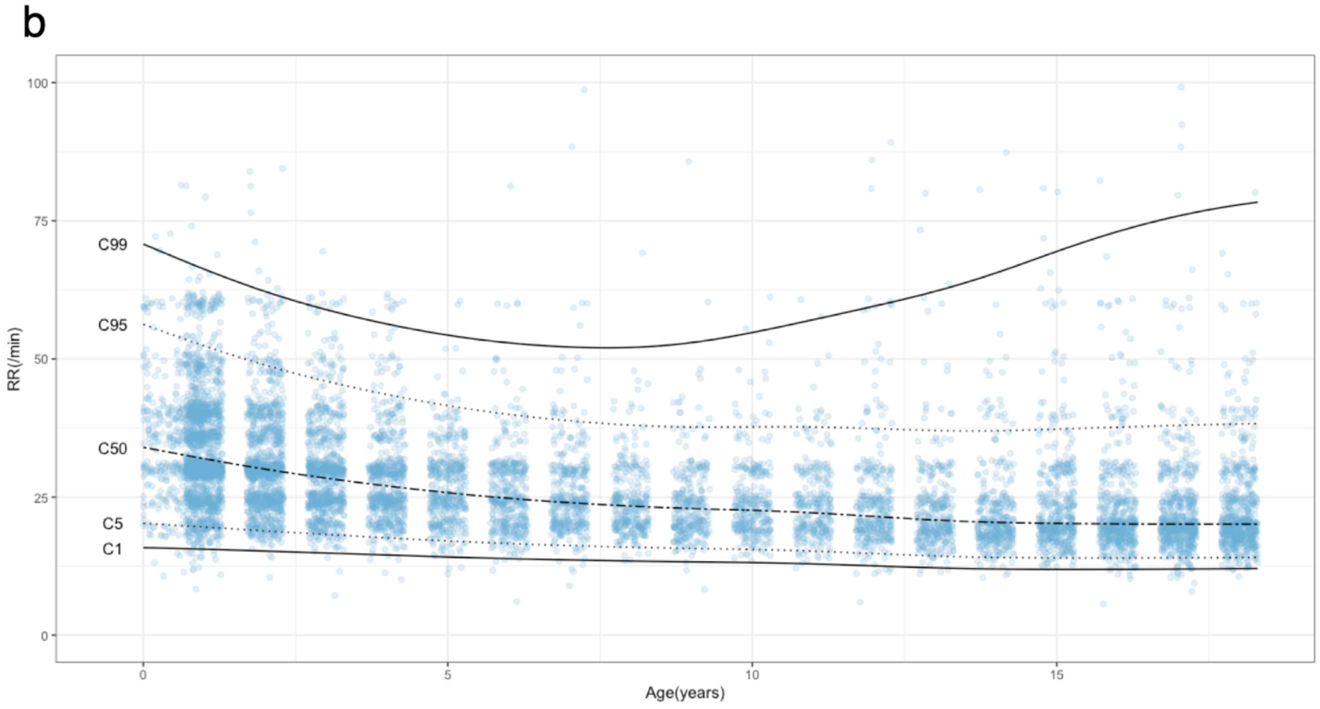

Development of centile curves

Since there may be evidence of digit preference in the RR data, we added normally distributed random noise with a mean of 0 and a variance of 1 to the RR data. We developed centile curves of the vital signs according to the patient age using the Box-Cox power exponential (BCPE) distribution in the generalized additive model

for location, scale, and shape (GAMLSS) package for R software program, version 4.1.0 (R Foundation for Statistical Computing, Vienna, Austria). The GAMLSS model is a statistical model developed to overcome the limitations of generalized linear models and generalized additive models, which can provide flexible models to be fitted to the data. The use of GAMLSS models with the BCPE distribution has been instrumental in developing percentile curves for growth or vital signs. The use of GAMLSS models enabled us to determine the z-score of the vital signs at any point.

Next, by creating curves for the HRs and RRs with 10 bootstrapped samples, we determined the stabilities of the 1st and 99th percentiles by comparing the differences between those samples. We also validated the curves by dividing each bootstrapped dataset into five age groups of equal size and determining the proportion of the HRs and RRs below the 5th and above the 95th percentiles for each age group, both of which would be expected to be approximately 5%.

We calculated the mean values of the 1st, 5th, 10th, 50th, 90th, 95th, and 99th percentiles for the HRs and RRs within each of the 13 age groups, which were defined as cut-off points for clinical use. We used the same age groups that were used in previous studies, [1,2,4] to enable direct comparisons with those studies.

The stability and validation results are shown in the tables below.

Stability of the 1st and 99th centile values in 10 bootstrapped samples

Age group	Heart rate						Respiratory rate					
	1st centile			99th centile			1st centile			99th centile		
	Min	Mean	Max	Min	Mean	Max	Min	Mean	Max	Min	Mean	Max
0 m	76	81	89	204	212	217	14	15	17	68	72	77
3 m	79	83	88	205	211	214	15	15	16	67	71	74
6 m	81	84	87	206	209	212	15	15	16	66	69	71
9 m	83	85	87	206	208	211	15	15	16	65	67	69
12 m	83	85	86	204	206	208	15	15	16	65	66	67
18 m	83	85	88	197	200	203	15	15	16	62	64	65
2 y	82	84	86	191	195	197	15	15	16	60	62	63
3 y	79	81	83	183	185	188	15	15	15	57	59	60
4 y	76	77	79	174	177	180	14	15	15	54	56	57
5 y	73	74	76	169	172	174	14	14	15	53	54	57
6 y	68	70	71	166	167	168	13	14	14	52	53	56
7 y	63	66	67	159	162	165	13	14	14	51	52	55
8 y	61	63	65	155	158	163	13	14	14	49	51	54
9 y	60	62	63	155	156	159	13	13	14	49	52	55
10 y	58	60	62	152	154	157	13	13	14	51	55	59
11 y	56	58	60	147	150	153	12	13	14	56	59	68
12 y	55	57	58	145	148	152	12	13	13	57	63	76
13 y	55	56	57	145	146	148	12	12	13	58	65	76
14 y	54	55	56	144	145	148	12	12	13	60	66	72
15 y	53	55	56	143	145	148	11	12	13	62	69	74
16 y	53	54	55	141	144	146	11	12	13	64	73	78
17 y	52	53	54	140	142	145	11	12	13	66	75	80
18 y	53	54	55	141	144	146	12	12	12	68	76	81

m: months; Max: maximum; Min: minimum; y: years

Proportions of observations outside of the 5th and 95th centile values

	Heart rate						Respiratory rate					
	5th centile			95th centile			5th centile			95th centile		
	Min.	Mean	Max.	Min.	Mean	Max.	Min.	Mean	Max.	Min.	Mean	Max.
Age 1 (<1 y)	0.038	0.045	0.052	0.055	0.061	0.073	0.038	0.051	0.069	0.048	0.058	0.070
Age 2 (1 to <4 y)	0.037	0.042	0.047	0.035	0.050	0.056	0.036	0.040	0.045	0.051	0.057	0.067
Age 3 (4 to <8 y)	0.042	0.047	0.053	0.044	0.051	0.055	0.023	0.030	0.039	0.045	0.048	0.052
Age 4 (8 to <15 y)	0.051	0.056	0.059	0.040	0.050	0.059	0.018	0.027	0.031	0.053	0.059	0.067
Age 5 (≥15 y)	0.050	0.055	0.061	0.044	0.048	0.053	0.018	0.021	0.026	0.057	0.068	0.075

Max.: maximum; Min.: minimum; y: years

Supplementary Figure S4. Prehospital severity criteria for children and Trauma prehospital severity criteria of the Kobe city emergency transport system. The reference ranges of vital signs were defined by local experts.

1. Pediatric prehospital severity criteria

Step 1. Physiological evaluation (in case any of the following item is recognized)

Consciousness: Japan Coma Scale* ≥ 100

Breathing:

Neonate (under 28 days)	< 30/min or ≥ 50/min
Infant (0-1 year old)	< 20/min or ≥ 30/min
Child (1-6 years old)	< 20/min or ≥ 30/min
Adolescent (6-12 years old)	< 15/min or ≥ 30/min
Laterality of respiratory sound	
Abnormal breathing pattern	

Pulse:

Neonate (under 28 days)	< 100 bpm or ≥ 150 bpm
Infant (0-1 year old)	< 80 bpm or ≥ 120 bpm
Child (1-6 years old)	< 60 bpm or ≥ 110 bpm
Adolescent (6-12 years old)	< 60 bpm or ≥ 110 bpm

Systolic blood pressure:

Neonate (under 28 days)	< 70mmHg
Infant (0-1 year old)	< 80mmHg
Child (1-6 years old)	< 80mmHg
Adolescent (6-12 years old)	< 90mmHg

SpO2: <90%

Others: signs of shock, cool extremities, pallor, altered level of consciousness, poor perfusion with weak central pulses, tachycardia: >180/min, prolonged capillary refilling time: >2 sec

NO → Step 2

YES → Severe, critical or deceased
Transport to a tertiary or equivalent hospital

Step 2. Symptoms

Persistent seizure more than 20 min, repeated vomiting, hypothermia, dehydration, unusual irritability, agitation, abnormal pupils (mydriasis or miosis), bleeding diathesis

NO → Mild or moderate

YES → Severe, critical or deceased
Transport to a tertiary or equivalent hospital

*** Japan Coma Scale**

0: Clear.
1: Almost fully conscious.
2: Unable to recognize time, place, and person.
3: Unable to recall name or date of birth of oneself.
10: Arousable by being spoken to but reverts to the previous state if stimulus stops.
20: Arousable with a loud voice but reverts to the previous state if stimulus stops.
30: Arousable only by repeated mechanical stimuli.
100: Unarousable using any forceful stimuli but responds to avoid the stimuli.
200: Unarousable using any forceful stimuli but responds with slight movements, including decerebrate or decorticate postures.
300: Unarousable using any forceful stimuli and does not respond at all.

Supplementary Figure S4 (continued)

2. Trauma prehospital severity criteria (common for both adults and children)

Step 1. Physiological evaluation (in case any of the following item is recognized)

Consciousness: Japan Coma Scale ≥ 20
Breathing:
< 10/min or ≥ 30/min
Laterality of respiratory sound
Abnormal breathing pattern
Pulse: < 50 bpm or ≥ 120 bpm
Systolic blood pressure: < 90mmHg or ≥ 200mmHg
SpO2: < 90%
Others: signs of shock

NO → Step 2

YES → Severe, critical or deceased
Transport to a tertiary or equivalent hospital

Step 2. Anatomical evaluation
Fractures of facial bones, cervical or thoracic pneumohypoderma, flail chest, distension of the externa jugular vein, abdominal swelling, muscular defense, pelvic fracture, both femoral fracture, penetrating injury to head/thorax/abdomen /cervix/inguinal region, burns with 15% to total body surface area, airway burns, facial burns, degloving injury, multiple finger amputations, amputation of an extremity, paralysis of an extremity.

NO → Step 3

YES → Severe, critical or deceased
Transport to a tertiary or equivalent hospital

Step 3.Situation
Passenger's death, thrown out of a car, run over by a car, hit by a car for more than 5m, severe damage to the car, more than 20 min for evacuation, car rollover, distance between the fallen motorcycle and the driver: long, a car collides with a pedestrian/bicycle, caught in machinery, the trunk was involved, fallen from a high place

NO → Mild or moderate

YES → Severe, critical or deceased
Transport to a tertiary or equivalent hospital

The definition of severity judged by emergency medical service providers
- Mild: the emergency medical service provider does not expect hospitalization
- Moderate: the emergency medical service provider expects hospitalization but not severe
- Severe: with life in danger
- Critical: impending life crisis (cardiac arrest, respiratory arrest, requiring cardiopulmonary resuscitation)
- Deceased: death at the time of contact
